# Exploring the microbial diversity and characterization of cellulase and hemicellulase genes in goat rumen: a metagenomic approach

**DOI:** 10.1186/s12896-023-00821-6

**Published:** 2023-12-04

**Authors:** Santosh Thapa, Suping Zhou, Joshua O’Hair, Kamal Al Nasr, Alexander Ropelewski, Hui Li

**Affiliations:** 1 Department of Agricultural and Environmental Sciences, College of Agriculture, Tennessee State University, 3500 John A. Merritt Blvd, Nashville, TN 37209 USA; 2 Vanderbilt University Medical Center, 2215 Garland Ave, Nashville, TN 37232 USA; 3 Department of Biological Sciences, College of Life & Physical Sciences, Tennessee State University, 3500 John A. Merritt Blvd, Nashville, TN 37209 USA; 4 Department of Computer Sciences, College of Engineering, Tennessee State University, 3500 John A. Merritt Blvd, Nashville, TN 37209 USA; 5 Pittsburgh Supercomputing Center, 300 S. Craig Street, Pittsburgh, PA 15213 USA

**Keywords:** Goat rumen bacteria, Metagenome/ shotgun sequencing, De novo assembly, Gene annotation, Rumen microbial ecology, Cellulolytic/ xylanolytic gene

## Abstract

**Background:**

Goat rumen microbial communities are perceived as one of the most potential biochemical reservoirs of multi-functional enzymes, which are applicable to enhance wide array of bioprocesses such as the hydrolysis of cellulose and hemi-cellulose into fermentable sugar for biofuel and other value-added biochemical production. Even though, the limited understanding of rumen microbial genetic diversity and the absence of effective screening culture methods have impeded the full utilization of these potential enzymes. In this study, we applied culture independent metagenomics sequencing approach to isolate, and identify microbial communities in goat rumen, meanwhile, clone and functionally characterize novel cellulase and xylanase genes in goat rumen bacterial communities.

**Results:**

Bacterial DNA samples were extracted from goat rumen fluid. Three genomic libraries were sequenced using Illumina HiSeq 2000 for paired-end 100-bp (PE100) and Illumina HiSeq 2500 for paired-end 125-bp (PE125). A total of 435gb raw reads were generated. Taxonomic analysis using Graphlan revealed that *Fibrobacter*, *Prevotella*, and *Ruminococcus* are the most abundant genera of bacteria in goat rumen. SPAdes assembly and prodigal annotation were performed. The contigs were also annotated using the DOE-JGI pipeline. In total, 117,502 CAZymes, comprising endoglucanases, exoglucanases, beta-glucosidases, xylosidases, and xylanases, were detected in all three samples. Two genes with predicted cellulolytic/xylanolytic activities were cloned and expressed in *E. coli* BL21(DE3). The endoglucanases and xylanase enzymatic activities of the recombinant proteins were confirmed using substrate plate assay and dinitrosalicylic acid (DNS) analysis. The 3D structures of endoglucanase A and endo-1,4-beta xylanase was predicted using the Swiss Model. Based on the 3D structure analysis, the two enzymes isolated from goat’s rumen metagenome are unique with only 56–59% similarities to those homologous proteins in protein data bank (PDB) meanwhile, the structures of the enzymes also displayed greater stability, and higher catalytic activity.

**Conclusions:**

In summary, this study provided the database resources of bacterial metagenomes from goat’s rumen fluid, including gene sequences with annotated functions and methods for gene isolation and over-expression of cellulolytic enzymes; and a wealth of genes in the metabolic pathways affecting food and nutrition of ruminant animals.

**Supplementary Information:**

The online version contains supplementary material available at 10.1186/s12896-023-00821-6.

## Background

Over the past two decades, there has been an increased global interest in the development of sustainable bio-renewable primarily owing to the increase in greenhouse gas emission, climate change and ultimately to reduce the dependency on fossil fuels [1]. Cellulosic biomass without any doubt is emerging as a sustainable raw material for the bioeconomy. Lignocellulosic biomass comprises mainly of polysaccharide polymers, cellulose, hemi-cellulose, pectin, and lignin [2]. The incorporation of enzymatic synthesis into a wide array of eco-friendly bioprocesses such as the hydrolysis of cellulose/hemi-cellulose has become an illustrious tool in deriving well defined bioactive compounds and biodegradable industrial products. Yet, the potential exploitation of cellulosic biomass conversion into its oligo and monosaccharides is particularly hindered due to the limited understanding of the complex recalcitrant nature of cellulose, hemi-cellulose and lignin, distinct biochemical functions of the enzyme, enzymatic pathways, and the dearth of tailor-made suitable efficient enzymes [3, 4]. This led to the increased investigation of novel hydrolytic enzymes from unique and extreme ecological niches. Hence, it is of utmost significance to understand the phenomenon behind the unexploited ecologically sustainable microbial bioresource.

The various kinds of cellulolytic and xylanolytic enzymes are found in microbes, plants, snails, termites, beetles, insects dwelling in various extreme environmental niches. Microorganisms are the prime producers of cellulolytic and xylanolytic enzymes which makes them the most prominent players in biomass decomposition [5]. Chen et al. reported that microbial enzymes possess the remarkable capability to significantly expedite the otherwise highly protracted process of biodegrading cellulosic biomass [6]. Ruminant’s rumen houses dense and complex community of symbiotic microbes that work together to break down lignocellulose [7]. These rumen microbial communities are perceived as the most potential biochemical reservoir of inordinately diverse and multi-functional cellulolytic enzymes with peculiar functional adaptation to enhance green biotechnological processes [8]. Bacterial community dominates the ruminal environment and hence considered as the most efficient biomass degrading enzymes in the herbivore gut microbiome. Despite this fact, the infancy in understanding about the rumen microbial genetic diversity and a lack of suitable screening culture techniques has limited the exploitation of multiple promising enzymes. To date, less than 5% of the microorganisms on Earth are being cultivated using traditional laboratory techniques (i.e., great plate count anomaly) [9]. Owing to this documented disparity between cultivable and in situ diversity, a huge biodiversity of microbial community is inevitably misread. The recent advancement of metagenomics strategy has obtained great popularity for the culture free recovery of near complete microbial genomes from complex environmental niches.

With the development of metagenomics, meta-transcriptomic and metaproteomic, numerous studies of the gut microbiome of wood feeding insects, termites, ruminant animals (horses, cattle) have been reported with the discovery of diverse cellulolytic enzymes [10–15]. In 2011, Hess et.al reported that only 0.03% of the assembled rumen metagenome had hits to sequenced organisms [16]. Since then, thousands of bacterial metagenomes have been sequenced and deposited into public repositories. In 2018, Stewart et al. assembled 913 draft bacterial and archaeal Metagenome-Assembled Genomes (MAGs) from an extensive dataset of rumen metagenomic sequences obtained from 43 Scottish cattle [17]. In the work conducted by Seshadri et al., they introduced the Hungate1000 collection, which comprises 410 culturable archaeal and bacterial genomes. Remarkably, their analysis revealed that 336 of these organisms were detected in rumen metagenomic datasets [18]. In their comprehensive analysis, Li et al. uncovered 13,825,880 non-redundant bovine rumen prokaryotic genes, notably dominated by functional species specializing in the degradation of plant cell wall materials and methane production [19]. Variation in diet, morphology, physiology substrate availability and genetic makeup results diversity in the GIT (gastrointestinal tract) microbiomes. In a recent comparative metagenomics investigation of rumen ecosystems, conducted by Glendinning et al., a total of 391 MAGs were constructed across various ruminant species, including cows, buffaloes, sheep, and reindeer. This study unveiled substantial distinctions in ruminal microbiomes, as evidenced by variations in taxonomic composition and the presence of CAZymes genes [20]. In a separate investigation conducted by Han et al., the study delves into the influence of rumen degradable starch (RDS) levels on gut microbiota diversity and carbohydrate-active enzymes (CAZymes) in dairy goats. Their findings underscore that a high RDS diet is correlated with gastrointestinal health concerns, including inflammation, mucosal damage, and changes in gene expression [21]. Concurrently, investigations employing 16S rRNA analysis to investigate the phylogenetic diversity and community structure of African ruminants, yaks, deer, sheep, cattle, and reindeer have consistently revealed the significant influence of both diet and host genotype in shaping the composition and traits of the rumen microbiome [22–25]. Even so, metagenomic sequences from the rumen continue to yield novel and unique sequences that are distinct from those found in public databases [26]. Moreover, only limited works have reported the cloning of genes encoding glycosyl hydrolases inhabiting goat rumen bacterial metagenomes and their diversity and metabolic functions with respect to cellulosic biomass degradation. In this study, we encompass an analysis of goat rumen bacterial diversity exploiting a sequence driven metagenomic approach. Furthermore, the potential candidate genes encoding for cellulolytic and xylanolytic enzymes were further cloned and expressed to perform biochemical characterization of enzyme functionality.

## Methods

### Rumen sample collection and metagenomic DNA isolation

Rumen fluid was obtained from eight 1–2-year-old male meat goats when they were slaughtered at the goat farm at Langston University, Oklahoma. Goats were fed on a natural diet and hay [27]. (The rumen fluid was provided by Dr. Puchala at Langston University; no live animals were used in the study.) The rumen fluid was filtered through three layers of cheese cloth. Filtrates were used to extract genomic DNA following using the reagents for bacterial DNA extraction in FastDNA SPIN Kit (MP Biomedical, LLC, Solon, Ohio, USA) with modifications. Genomic DNA was purified further using the GeneClean Spin Kit (MP Biomedical). DNA concentrations were quantified with NanoDrop ND-1000 spectrophotometer (Thermo-Fisher, CA, USA). The quality of DNA (integrity) was confirmed by analysis on 1.0% agarose gel. The extracted genomic DNA was stored at -20 °C until further use.

### Metagenomic DNA sequencing, assembly and annotation

For DNA sequencing, approximately 0.1 µg of the metagenomic DNA sample was used to construct the sequencing library using Nextera DNA Sample prep kits (Illumina, San Diego, CA). The resulting libraries had a range of fragments from approximately 200–400 bp and were quantified using a Qubit spectrofluorometer (Invitrogen, CA). Three libraries were prepared from goat rumen metagenomics DNA samples namely Bct_789, Bct_5121, and Bct_5122. The Bct_789 library was sequenced on an Illumina HiSeq 2000 using TruSeq SBS kit v3 for paired end 100 bp sequencing; the Bct_5121 and Bct_5122 libraries were sequenced on an Illumina Hiseq 2500 for paired end 125 bp sequencing respectively (Genomics Facility, Cornell University). The three libraries generated a total of 435 gb reads. The raw reads were deposited in the NCBI Sequence Read Archive (SRA) under accession number SRX2267715 for Bct_789, and SRX2267714 for Bct_5121, and Bct_5122.

The raw reads were processed using Cutadapt 4 program, which include trimming, filtering (-q 15,15 –trim-n -m 31 –pair-filter = any) and removal of adapter sequences (-b CAAGCAGAAGACGGCATACGAGATCTAGTACGGTCTCGTGGGCTCGG). The resultant high-quality reads were assembled using three kmer sizes (-k 35, 55, 75) in SPAdes [28, 29]. Annotation of the SPAdes assemblies was using the Prodigal gene prediction programs and Diamond searches against UniProt Bacterial sequences (only the top matching in each scaffold was listed) [30, 31]. Metaphlan and Graphlan were used to produce a phylogenetic classification across all three datasets [32]. All the computational analysis were completed by using pipeline from the Pittsburgh Blacklight Supercomputer (Pittsburgh Supercomputing Center, Pittsburgh, PA, https://www.psc.edu/).

### Phylogenetic taxonomy and functional gene classification

The high-quality reads from Bct_789 were also subjected to Velvet (kmer size = 79) and SSPACE followed by CAP3 for assembly [33]. The assembled scaffolds were submitted to DOE-JGI for the Metagenome Annotation Pipeline (MAP v4) [34]. This annotation process encompasses the prediction of various elements, including CRISPR elements, non-coding and protein-coding genes. Briefly, the CRT and PILER-CR v1.06 tools were used for CRISPR element identification; a combination of Hidden Markov Models (HMMs) and ab initio gene calling algorithms was used for protein-coding genes and non-coding RNA genes identification; tRNAscan SE-1.3.1 was employed for tRNA prediction; hmmsearch tool from HMMER 3.1b2 was used for ribosomal RNA genes (5S, 16S, 23S) prediction; a consensus approach that combines the results of four ab initio gene prediction tools prokaryotic GeneMark.hmm (v. 2.8), MetaGeneAnnotator (v. Aug 2008), Prodigal (v. 2.6.2), and FragGeneScan (v. 1.16) was used for protein-coding gene prediction. Functional annotation is performed by associating protein-coding genes with Clusters of Orthologous Groups (COGs) employing RPS-BLAST 2.2.31. Based on the COG classification in DOE-JGI Integrated Microbial Genomes (IMG, https://img.jgi.doe.gov/), the annotated genes were classified into 26 COG functional categories. Putative endo-glucanase, exo-glucanase, and beta-glucosidase for cellulose degradation, endo-beta xylanase, beta-xylosidase for hemi-cellulose degradation were retrieved from carbohydrate transport and metabolism group of genes (279,864 gene count annotated).

### Gene cloning with TOPO cloning system

The gene-specific primers for cellulase and hemi-cellulase genes were designed using OligoPerfect TM Designer (https://tools.lifetechnologies.com/content.cfm?pageid=9716, Table S1). In total, 14 cellulase and hemicellulase genes were cloned from the goat’s rumen metagenomic DNAs. PCR products were separated on 0.7% agarose gels. The DNA fragments of expected sizes were excised and purified from the gel using the QIAquick Gel Extraction Kit (Cat. No. 28704). The amplified genes were subsequently ligated to pET101 vector (Invitrogen, CA) and transformed into *E. coli* TOP10. Sanger sequencing was used to confirm the sequences of cloned gene. After the confirmation of 100% identity, these cloned sequences were submitted to the NCBI databank (http://www.ncbi.nlm.nih.gov).

### Recombinant protein over-expression and characterization

The pET101 plasmids containing full-length open reading frames of the cloned genes were transformed into *E.coli* BL21 (DE3). The overnight BL21 culture was inoculated into LB with ampicillin and incubated for 2–3 h at 37 °C with agitation at 200 rpm until the culture's absorbance OD600 = 0.6–0.8. At this point, 0.6 mM IPTG was added to induce protein expression. Following a subsequent 5–6 h of post-induction at approximately 37 °C, the cells were harvested through centrifugation at 6,000X g for 5 min. To obtain crude protein, the cells were resuspended in a 100 mM HEPES buffer (pH 7.5) and subjected to sonication with three 30-s bursts separated by 1-min intervals, utilizing an amplitude of 65%. Crude protein samples were then mixed with 2X Laemmli Sample Buffer (BioRad) containing 5% β-mercaptoethanol. The protein samples were separated on a 10–20% sodium dodecyl sulfate polyacrylamide electrophoresis (SDS-PAGE) gel. The molecular weight (size) of the proteins was confirmed using Colloidal Blue Staining (Invitrogen).

To confirm the enzymatic activity of the recombinant proteins, fresh bacterial culture was directly inoculated into assay plates containing suitable substrates: carboxymethyl cellulose sodium salt (CMC) for endoglucanase and xylan for endo-1,4-beta xylanase. Then the plates were incubated at 37 °C for next 48 h. After incubation, the cellulolytic/xylanolytic activities were assayed using the Congo Red staining method [35, 36].

To quantify the enzymatic activity, freshly grown bacteria were lysed. And supernatant containing crude protein was tested for its ability to hydrolyze CMC, and xylan oat spelt, a substrate for the activity assay of endoglucanase A and endo-1,4-beta xylanase respectively. The reducing sugar released upon the hydrolysis of sugar polymers was determined using 3,5-dinitrosalicyclic acid (DNS) method [37]. The reducing sugar content was measured spectrophotometrically at 540 nm (Milton Roy Spectrophotometer, Model 601). One unit of enzymatic activity was defined as the amount of enzyme that liberates 1 μmol of reducing sugar from the substrate per minute under the above-mentioned assay conditions.

To determine the optimum pH, the recombinant crude enzyme was incubated at 50 °C for 45 min at pH 4.0–6.0 (sodium acetate buffer), pH7.0–8.0 (sodium phosphate buffer) and pH 9.0–10.0 (Tris–HCl buffer). The optimum temperature for endoglucanase A was determined by assessing enzyme activity in the range of 20–70 °C using CMC at 1% following a 45-min incubation at pH 6.0 (in a sodium acetate buffer). The same approach was employed for endo-1,4-beta xylanase, with the only adjustment being the pH set to 10.0 (in a Tris–HCl buffer). The pH stability was determined after keeping the enzymes at different pH at 50 °C for 24 h. The temperature stability was analyzed following the pre-incubation of endoglucanse A at pH-6.0 and endo-1,4-beta xylanase at pH-10.0 within a temperature range of 20–70 °C for 1 h respectively before further enzymatic activity test [38, 39].

### Domain analysis and homology modeling of endoglucanase A and endo-1,4-beta xylanase

The analysis of protein domain architecture was performed using SMART program (http://smart.embl-heidelberg.de/). For phylogenetic analysis, 26 endoglucanase genes and 25 endo-1,4-beta xylanase genes were selected respectively from the NCBI, CAZY, UniProt and PDB databases [40], which cover a range of microorganism from bacteria, fungi, archaea, virus and unclassified organisms. The hit sequences were then aligned using neighbor-joining algorithm and P-distance model with the bootstrap simulation in MEGA X [41]. Furthermore, bootstrapping with resampling method of Felsenstein and 1000 bootstrap replicates was done in order to examine the robustness of the phylogenetic tree topology [42].

Multiple sequence alignments of the target proteins (endoglucanase A/ endo-1,4-beta xylanase) against their selected homology proteins were performed with the Clustal Omega software (https://www.ebi.ac.uk/Tools/msa/clustalo/). Furthermore, the sequence similarities and structural information from the aligned protein sequences were rendered through ESPript 3.0 analysis software package (http://espript.ibcp.fr/ESPript/cgi-bin/ESPript.cgi).

The tertiary structures of endoglucanase A and endo-1,4-beta xylanase was predicted using homology modeling in Swiss Model (https://swissmodel.expasy.org/) [43]. In total, 50 different template structures available in protein data bank (PDB) were tested as template for the 3D model of endoglucanase A. The template used for the prediction of the 3D structure of the recombinant endoglucanase A is the available crystal structures of ligand bound PbGH5A (glycoside hydrolase; PDB ID: 5D9N) from *Prevotella bryantii* (PbGH), which shares about 44% amino acid sequence identity [44]. Similarly, the template used to generate the homology model of endo-1,4-beta xylanase is an available crystal structure of endo 1, 4 beta D-Xylanase 10B (Xyn10B) (PDB ID: 2WYS) from *Clostridium thermocellum*.

To validate the predicted 3D structure from SWISS-MODEL, Ramachandran plot was analyzed using RAMPAGE. The predicted structure was analyzed based on the global model quality estimation (GMQE) score. The accuracy of the predicted model of endoglucanase A with GMQE score of 0.71 was evaluated by Ramachandran plot using the RAMPAGE server (http://mordred.bioc.cam.ac.uk/~rapper/rampage.php) [45]. The information about the fitness and validation of the predicted recombinant protein model was further confirmed using the Verify3D (http://servicesn.mbi.ucla.edu/Verify3D/). Meanwhile, secondary structure of the predicted model of the enzyme was determined by using online available server STRIDE (http://webclu.bio.wzw.tum.de/cgi-bin/stride/stridecgi.py) [46]. The prediction of salt bridges (distances ≤ 3.2A) was performed using visual molecular dynamics (VMD, version 1.9.3) [47].

## Results

### Metagenomic DNA assembly, microbial taxonomy and Diamond annotation

As shown in Table 1, the raw reads generated are 456,435,541*2 for Bac_5122, 176,691,539*2 for Bct_5121, and 216,313,953*2 for Bct_789. In total, there were 1,698,882,066 raw reads generated from the sequencing libraries. The high-quality reads generated using Cutadapt include: 439,742,222*2 in Bct_5122 sample, 174,079,701*2 in Bct_5121 sample, and 205,548,701*2 in Bct_789 sample. Three different kmer sizes including 35, 55, and 75 were used in SPAdes assembly, and kmer = 75 results in the best assembly scaffolds. The total number of scaffolds in Bct_5122 was 9,329,048, in Bct_5121 was 5,253,641, in Bct_789 was 4,842,139. The largest scaffolds were around 200,000–220,000 bp, and the number of scaffolds with length over 20,000 were around 6,000 in all three samples.

**Table 1:** Goat rumen bacterial (Bct) metagenome sequencing assembly

Datasets	Size (bp)	Raw reads*2 (PE)	Reads after filtering	Total No. of scaffolds	Largest Scaffold	No. of scaffolds > 20,000
Bct_5122	125	456,435,541	439,742,222	9,329,048	201,713	2,101
Bct_5121	125	176,691,539	174,079,701	5,253,641	222,488	1,946
Bct_789	100	216,313,953	205,548,701	4,842,139	212,207	1,951

**Fig. 1:**
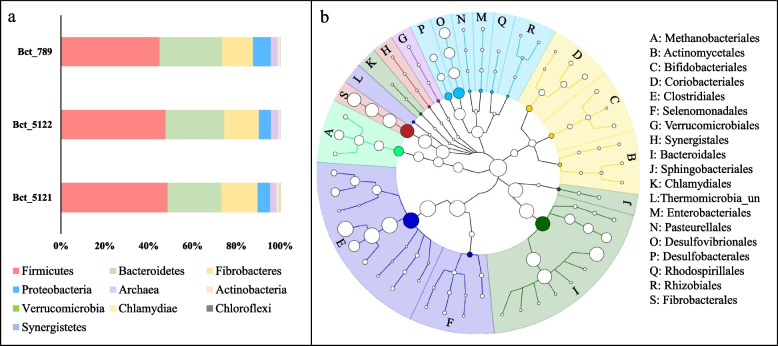
Goat rumen microbial community analysis using Metaphlan and Graphlan. **a** Approximately 96.6 -97.2% of fragments were assigned to bacteria, and 2.8–3.4% belonged to Archaea. The major phylum were *Firmicutes* (45–48.7%), *Bacteroidetes* (24.5–28.5%), and *Fibrobacteres* (14.1–16.5%). **b** The microorganisms annotated in all three datasets were combined. In total, 18 bacterial orders and 1 archaeal order comprised with 40 species were identified

Metaphlan and Graphlan were used to perform phylogenetic classification across the three datasets generated from the three libraries (Fig. 1). Bacterial taxonomic profiling indicated that at phylum level, *Firmicutes*, *Bacteroidetes*, and *Fibrobacteres* were the dominant bacteria presenting in the goat rumen, which accounts for around 90% in total among others (Fig. 1a). In total, there were 18 bacterial orders and 1 archaeal order comprised with 40 species that were detected in goat rumen samples (Fig. 1b). At species level, *Mathanobrevibacter_unclassified* (2.75%, Archeae) was the major Archeae species; *Butyrivibrio_unclassified* (37.8%, *Clostridiales*), and *Prevotella ruminicola* (22.7%, *Bacteroidales*), *Fibrobacter succinogenes* (15.5%, *Fibrobacterales*), *Butyrivibrio proteoclasticus* (5.8%, *Clostridiales*), *Desulfovibrio desulfuricans* (5%, *Desulfovibrionales*), *Bacteroides_unclassified* (3.5%, *Bacteroidales*) *Ruminococcus albus* (2%, *Clostridiales*) were the predominant bacterial species present in goat’s rumen (Supplement Table S2). Some of these bacterial species were also identified as the chief producers of CAZymes in goat’s rumen ecosystem for cellulose degradation. Archaea *Methanobacteria*, which belong to methane producing ruminal Methanogens were also identified in the assembled sequences.

Diamond searching against UniProt Bacterial sequences was performed on all the three datasets (only the top matching in each scaffold was listed). There were 3,334,049, 5,437,719, and 7,901,185 genes identified in Bct_789, Bct_5121, and Bct_5122 distinctively. In all three samples, a total of 19,780 glucanase and 43,692 beta-glucosidase genes were detected, indicating the high abundance of enzymes involved in cellulose degradation. Similarly, 20,881 xylanase and 18,295 xylosidase genes were identified, indicating the high capability of goat rumen bacteria in hemicellulose/xylan degradation. Additionally, 1,123 pectin methylesterase, 7,779 pectate lyase, and 5,852 polygalacturonase genes were identified, suggesting the potential for pectin degradation (Table 2). Across all three datasets, a total of 3,327 bacterial strains were annotated with genes involved in the degradation of cellulosic biomass. Among these strains, a higher proportion were detected to possess cellulase genes, while a smaller number had genes involved in pectin degradation. Notably, a combined total of 327 bacterial strains were found to harbor functional genes for the degradation of plant fiber (comprised with cellulose, xylan, and pectin). The analysis revealed the presence of all seven enzymes (mentioned above) in eight bacterial species, namely *Bacteroidals bacterium*, *Bacteroides sterorirosoris*, *Butyrivibrio proteoclasticus*, *Butyrivibrio sp* INIIa14, *Butyrivibrio sp* Su6, and three species of *Prevotella*.

**Table 2:**
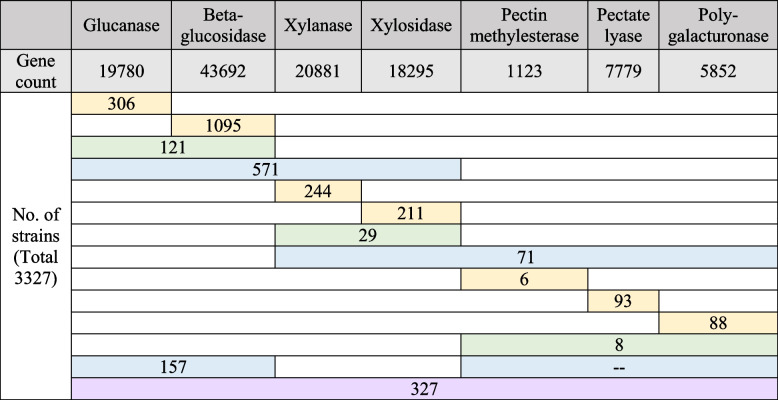
Gene counts for genes in cellulose, hemicellulose and pectin degradation using Diamond annotation

### DOE_JGI annotation

The assembled scaffolds of Bct_789 were submitted to the Integrated Microbial Genomes (IMG) for annotation with the Img taxon object ID # 3300001425. There were 10,024,714 sequences subjected to annotation analysis. The annotation detected 748 CRISPR counts, 2,261 16 s rRNA, and 10,276,848 protein coding genes (accounts for 99.86% of annotated sequences), out of which, 3,054,241 of the genes belong to the Cluster of Orthologous Groups (COG, 30% of annotated sequences) and 2,236,087 genes were placed under the Pfam protein family domains (Table 3).

**Table 3:** Statistics of the assembled sequences annotation using DOE-JGI pipeline

	**Number**	**% of Assembled**
**Number of sequences**	10024714	100.00%
**CRISPR Count**	748	
**Genes**		
RNA genes	14680	0.14%
rRNA genes	8545	0.08%
16S rRNA	2261	0.02%
23S rRNA	5291	0.05%
tRNA genes	6135	0.06%
Protein coding genes	10276848	99.86%
with COG	3054241	29.68%
with Pfam	2236087	21.73%
**COG Clusters**	4589	99.09%
**Pfam Clusters**	6355	33.14%

**Fig. 2:**
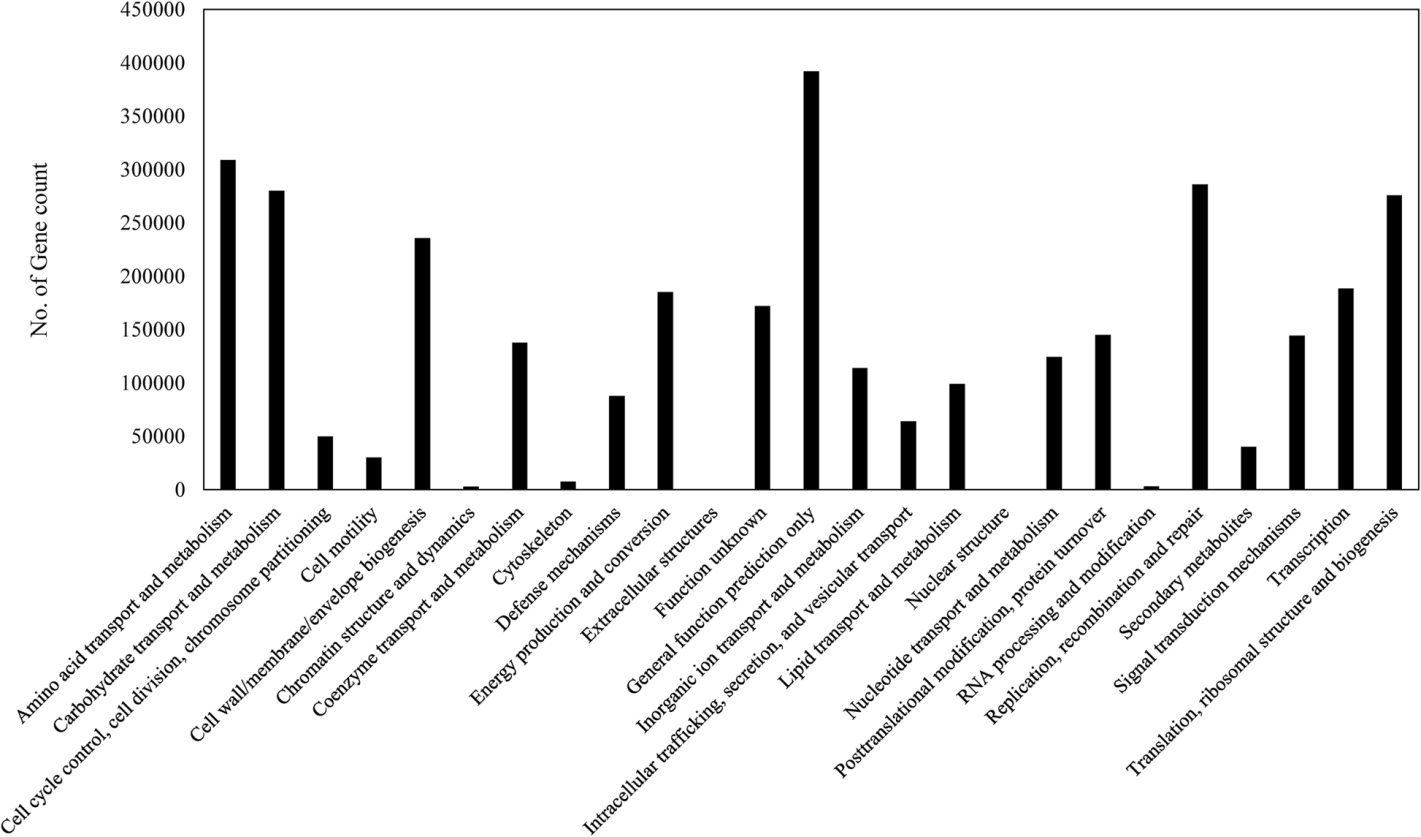
COG categories in DOE-JGI annotation

The 26 COG categories include general function prediction (11.62% of gene count), amino acid transport and metabolism (9.16% of gene count), carbohydrate transport and metabolism (8.3% of gene count), replication, recombination and repair (8.48% of gene count) and translation, ribosomal structure and biogenesis (8.18% of gene count), and cell wall/membrane/envelope biogenesis (6.98% of gene count). The total gene count for carbohydrate transport and metabolism was 279,864, which was the database to screen CAZymes for fiber digestion (Fig. 2, Table S3).

In goat’s rumen, the degradation of plant fibers is performed under the action of microbial enzymes. For the cellulolytic genes, carbohydrate transport and metabolism GO category includes 347 endo-1,4-beta-D-glucanase genes (COG3405), 14 exo-beta-1,3-glucanase genes (COG5309), 1579 beta-glucosidase genes (COG2723) and 26 cellobiase genes (COG5297) for cellulose degradation; 3115 alpha-L-arabinofuranosidase genes (COG3534), 1753 endo-1,4-beta xylanase genes (COG3693), peptidoglycan/xylan/chitin deacetylase (COG0726) and 4475 beta-xylosidase genes (COG3664/3507) for hemicellulose degradation; and 876 pectin methylesterase genes (COG4677), 2894 polygalacturonase genes (COG5434), 478 pectate lyase genes (COG3866) for pectin degradation.

Out of 10,276,848 protein coding genes, 3,054,241 genes were identified with matching COG categories. Those COG categories for amino acid transport and metabolism; and carbohydrate transport and metabolism; and replication, recombination and repair were annotated with the highest number of gene counts.

### Gene cloning and recombinant enzyme characterization

The five novel cellulase/xylanase genes namely endo-1,4-beta xylanase, endoglucanase A, beta-glucosidase A, endo-1,6 beta-D-glucanase, and endoglucanase E were cloned. These genes have been deposited in the NCBI GenBank databases under accessions KP851788, KP851789, KP851790, KP851791, and KP851792 respectively (Table S4). Two of the genes endo-1,4-beta xylanase and endoglucanase A were successfully transformed into *E. coli* BL21(DE3) and over-expressed with induction of IPTG. Proteins from cell lysates were separated on SDS-PAGE gel; the protein bands matched the expected molecular weight of the recombinant proteins, thus confirming the over-expression of the recombinant proteins in the bacterial clones (Fig. S1).

**Fig. 3:**
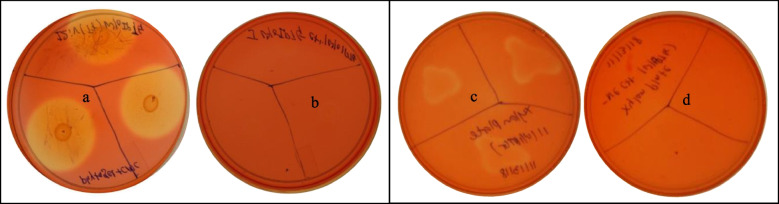
Plate enzymatic assay of endoglucanase A and endo-1,4-beta xylanase. **a**-**d** Plate assay determination of cellulolytic and xylanolytic activity by Congo red staining method; **a** and **c** were inoculated with BL21(DE3) harboring endoglucanase A and endo-1,4-beta xylanase respectively; **b** and **d** are negative control with inoculation of bacterial harboring empty vector

The activity of the recombinant enzymes was analyzed using the Congo red staining method. As shown in Fig. 3, the Congo red stained plates a and c (inoculated with recombinant bacterial colonies) exhibited a clear halo zone showing endoglucanase and endo-1,4-beta xylanase activities; on the two control plates (b, d) which were not inoculated with the bacterial inoculation, no substrate degradation was seen.

**Fig. 4:**
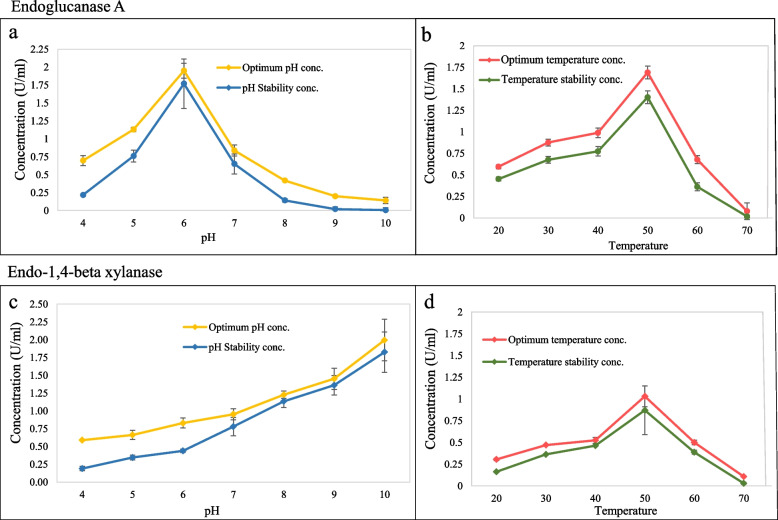
Enzymatic activity and stability of endoglucanase A and endo-1,4-beta xylanase. The optimized activity and stability of the recombinant endoglucanase A and endo-1,4-beta Xylanase were determined at different temperatures and pH values. **a** Effects of pH on the endoglucanase A enzyme activity at 50 °C. **b** Effects of temperature on the endoglucanase A activity within a temperature range of 20–70 °C, at pH 6.0. **c** Effects of pH on the endo-1,4-beta xylanase enzyme activity at 50 °C. **d** Effects of temperature on the endo-1,4-beta xylanase activity within a temperature range of 20–70 °C, at pH 10.0

The optimal enzyme activity of the crude recombinant endoglucanase A and endo-1,4-beta xylanase was analyzed at various pH and temperature. The optimum pH and temperature for endoglucanase A were pH 6.0 and 50 °C (Fig. 4a, b). The enzyme endoglucanase A displayed a higher thermostability which retaining above 50% of its activity at temperature 20–60 °C after 1-h incubation (Fig. 4b). However, its pH stability is relatively low, the enzyme activity at pH-4.0 and pH8.0–10.0 were severely decreased after incubation at 50 °C for 24 h (Fig. 4a). Within our testing range, the optimal enzymatic activity for endo-1,4-beta xylanase was observed at pH 10 and a temperature of 50 °C. However, it's worth noting that enzyme activity was not evaluated at pH levels greater than 10 (Fig. 4c, d). Similarly, the enzyme endo-1,4-beta xylanase retained over 50% activity at temperature ranging from 20–60 °C after 1-h incubation (Fig. 4d). Moreover, it retained about over 50% of its enzymatic activity at pH 5–10 after 24-h incubation at 50 °C (Fig. 4c).

### Sequence and phylogenetic analysis of endoglucanase A and endo-1,4-beta xylanase

**Fig. 5:**
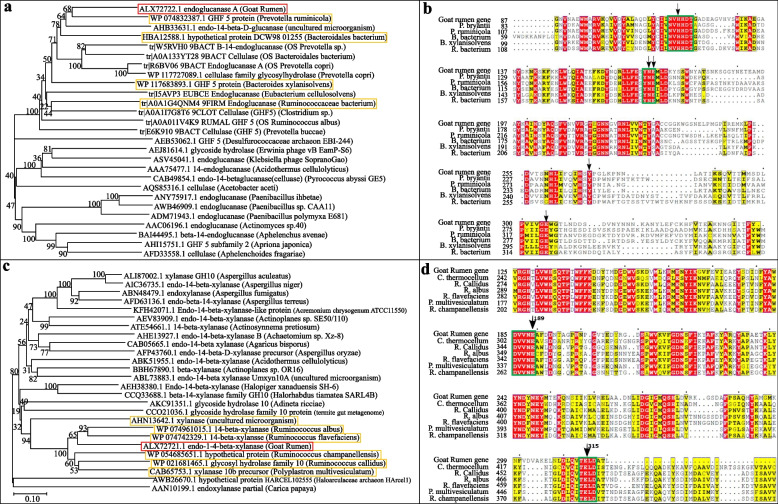
Phylogenetic and multiple alignment analysis of endoglucanase A and endo-1,4-beta xylanase. **a**, **c** Neighbor-joining phylogenetic tree of endoglucanase A and endo-1,4-beta xylanase based on protein sequences from various organisms. Scale bar corresponds to a genetic distance of 0.10 substitution/site. **b**, **d** Multiple alignments of the endoglucnase A domain (GrE) with other homologous protein GH5 domains and the endo-1,4-beta xylanase with other homologous protein GH10 domains

SMART protein sequence analysis stipulated that the putative enzyme endoglucanase A had a cellulase domain. A total of 26 endoglucanase protein sequences from the range of bacteria, fungi, Archaea, virus and unclassified organism were selected for the phylogenetic analysis. The phylogenetic analysis of endoglucanase A showed that it is closely related with the protein sequences from *Prevotella ruminicola* (WP074832387.1, Fig. 5a). Multiple alignments of the endoglucnase A with its homologous proteins in *Prevotella ruminicola* (WP074832387.1), *Bacteroidales bacterium* (HBA12588.1), *Bacteroides xylanisolvens* (WP117683893.1), *Ruminococcaceae bacterium* (A0A1G4QNM4) and *Prevotella bryantii* (PDB: 5d9M) indicated that the inferred amino acid sequence of a GH 5 family domain and the active site of the conserved domain (in green rectangle) with predicted catalytic residue (arrow pointed) in endoglucanase A were aligned with those of the homologous enzymes (Fig. 5b). Moreover, the protein shared less than 55.87% amino acid sequence identity with glycoside hydrolase family 5 protein from *Prevotella ruminicola* and 51.26% from *Alloprevotella* sp.

The SMART sequence analysis indicated that the endo-1,4-beta xylanase had a Glycosyl hydrolase-10 domain. Similarly, a total of 25 endo-1,4-beta xylanase proteins were selected for the neighbor-joining phylogenetic analysis. The analysis depicts the evolutionary relationship between endo-1,4-beta xylanase and associated proteins, which revealed that the target enzyme from goat rumen is closely related to the homology protein from *Ruminococcus albus* (WP074961015.1) and *Ruminococcus flavefaciens* (WP074742329.1, Fig. 5c). The schematic structure of multiple sequence alignment indicating that the inferred amino acid sequence of a GH 10 family domain in endo-1,4-beta xylanase was aligned with those of selected homologous enzymes from the following microorganisms: *Clostridium thermocellum* (PDB ID: 2WYS), *Ruminococcus callidus* (WP_021681465.1), *Ruminococcus albus* (WP_074961015.1), *Ruminococcus flavefaciens* (WP_074742329.1), *Polyplastron multivesiculatum* (CAB65753.1), *Ruminococcus champanellensis* (WP_054685651.1). Signature sequences (in green rectangle) with detection of predicted catalytic residue (black arrow) were well aligned among all samples (Fig. 5d). Moreover, the protein shared around 59% amino acid sequence identity with 1,4-beta xylanase glycosyl hydrolase family 10 protein from *Ruminococcus albus* and 57% amino acid sequence identity with 1,4-beta xylanase family 10 protein from *Ruminococcus flavefaciens* (WP074742329.1).

### Homology modelling and 3D structure prediction

**Fig. 6:**
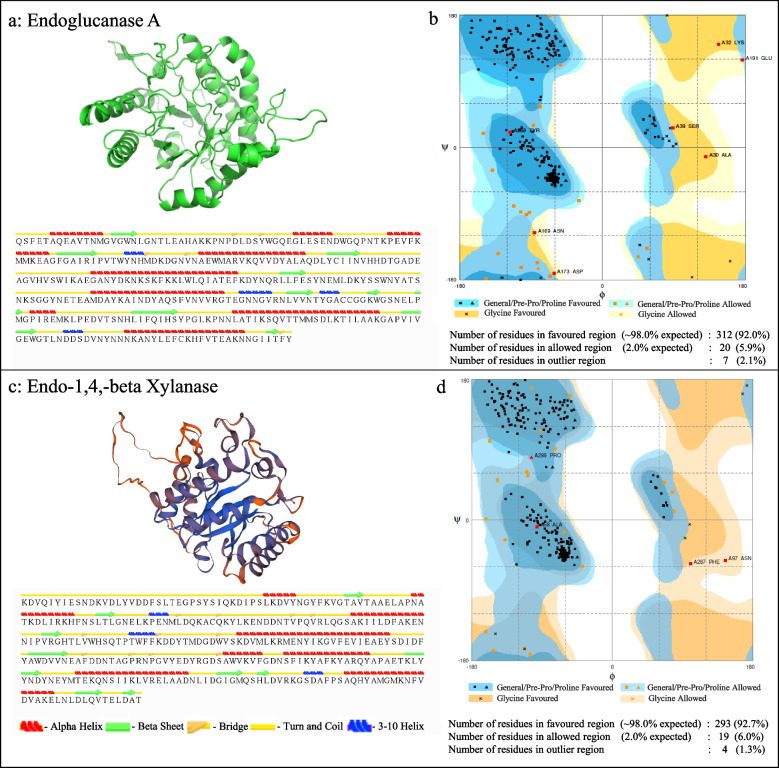
3D structure and overall composition analysis. Predicted 3D structure and overall composition (including alpha-helix, beta-sheet, bridge, turn and coil and 3–10 helix) of endoglucanase A (**a**), and endo-1,4-beta xylanase (**c**). Ramachandran plot analysis demonstrates the different residues falling in general favored (blue), general allowed (light blue), and glycine residues favored (yellow), glycine residue allowed (light yellow) for endoglucanase A (**b**), and endo-1,4-beta xylanase (**d**)

The tertiary structures of endoglucanase A and endo-1,4-beta xylanase are shown in Fig. 6a, c. Ramachandran plot indicates the quality and stereochemistry of the structure that identifies the torsion angles of the residues in favored regions, allowed regions and outliers. In the case of endoglucanase A, 92% of the residues had torsion angles in favored regions, 5.9% residues were in allowed regions and only 2.1% of the residues were the outliers (Fig. 6b). Similarly, for endo-1,4-beta xylanase, among 293 residues, 92.7% of the residues were in favored regions, 6% residues were in allowed regions and only 1.3% of the residues were in the disallowed regions (Fig. 6d).

STRIDE results suggested most of the secondary structures as coils and turns in the predicted protein structure. However, nine α-helices, ten β-strands and four 3_10_ helices were also present in the predicted structure of the recombinant endoglucanase A. Similarly, the secondary structure of endo-1,4-beta xylanase comprised of eight α- helices, nine β-strands and three 3_10_ helices along with coils and turns. In addition, the predicted model of the recombinant endoglucanase A and endo-1,4-beta xylanase was observed to be constituted of nineteen and thirteen salt bridges respectively (distances ≤ 3.2A).

## Discussion

Metagenome screening is an invaluable technique for exploring the vast biodiversity of nature and uncovering novel enzymes, as it allows for direct analysis without the limitations in cultivation-based methods. The mining of a metagenomic library has facilitated the identification of microbial diversity and novel enzymes (cellulase and xylanase) from a variety of environmental samples, including soil, hot spring, termite’s gut, rumen of dairy cow [48–51]. Earlier study identified that experimental warming and the resultant decrease in soil moisture has a significant impact on microbial biodiversity by reducing the richness of bacteria (9.6%). Furthermore, a recent study successfully mined the camel rumen metagenome to identify a novel alkali-thermostable xylanase that could enhance the conversion of lignocellulosic biomass [52].

The goat rumen is home to a diverse community of microorganisms, including bacteria, protozoa, and fungi, which collectively contribute to the digestion of fibrous plant materials and the extraction of essential nutrients [53]. These microbes are adept at breaking down complex carbohydrates, such as cellulose and hemicellulose, into simpler sugars and short-chain fatty acids through fermentation processes [54]. This breakdown not only provides goats with a vital source of energy but also aids in the absorption of nutrients, including proteins and vitamins. Moreover, the microbial population in the rumen helps maintain the pH balance, ensuring efficient digestion and preventing conditions like acidosis [55]. Bacterial population is the most abundant in the rumen ecosystem comprising 10^10^ to 10^11^ cells/ml [56]. Studies have shown that the composition and diversity of rumen microbes can be influenced by various factors, including diet, genetics, and environmental conditions, highlighting the intricate relationship between rumen microbiota and goat health and nutrition [57, 58]. Understanding and optimizing this microbial ecosystem is crucial for enhancing goat productivity and overall well-being. For this reason, there is an utmost need for the comprehensive exploitation of goat rumen bacterial population. The goats that were used to extract the ruminant fluids in this study were on diet rich in cellulose and xylan. Here, we utilized genome-centric metagenomics strategy to explore diverse phylogeny, cellulose degrading potential bacterial enzymes housed in goat rumen. We successfully identified 19,780 glucanase and 43,692 beta-glucosidase for cellulose degradation, 20,881 xylanase and 18,295 xylosidase genes for hemicellulose/ xylan degradation, and 1,123 pectin methylesterase, 7,779 pectate lyase, and 5,852 polygalacturonase for pectin digestion in 3,327 bacterial strains from goat rumen samples. Eight bacterial strains were identified with a full spectrum of enzymes for cellulosic biomass digestion including *Bacteroidals bacterium*, *Bacteroides sterorirosoris*, *Butyrivibrio proteoclasticus*, *Butyrivibrio sp INIIa14*, *Butyrivibrio sp Su6*, and three *Prevotella* species. Findings from this study clearly confirmed the rich containment of cellulolytic genes/enzymes and microbes in the goat’s rumen fluids. Our data concur with reports that the rumen microbiomes of browse-feed animals contain a high variety of glycoside hydrolases indispensable for degrading plant cell wall materials [59–62]. In this goat rumen sample, *Butyrivibrio proteoclasticus*, *Prevotella ruminicola,* and *Fibrobacter succinogenes* were identified as the predominant bacteria in the goat’s rumen microbiomes. These bacterial species are known for the ability to efficiently degrade and use cellulose as a carbohydrate source, which could be the primary microbes for fiber degradation in goats as well as other ruminant animals [63–65]. In addition, *Butyrivibrio proteoclasticus* previously known as *Clostridium proteoclasticum* demonstrated the ability to convert linoleic acid into stearic acid in sheep rumen, suggesting its significant role in lipid metabolism [66]. Delgado's study explored into the rumen microbiota and feed efficiency traits of Holstein cattle, shedding light on the fact that cattle with high feed efficiency had a heightened presence of *Bacteroidetes* and *Prevotella*. These results emphasize the critical role played by microbiota composition in influencing feed utilization performance [67]. In research assessing the impact of hainanmycin (HAI) and monensin (MON) supplementation on ruminal protein metabolism and the populations of proteolytic bacteria in Holstein heifers, a notable increase in the abundance of *Prevotella ruminicola* was detected. This finding underscores the significant role these bacteria play in protein metabolism [68]. Given that the productivity of meat and milk relies heavily on the microbiota's efficiency in breaking down plant cell walls, and conversion into protein and lipids, the recognition of key rumen microbiota assumes a pivotal role in shaping strategies aimed at optimizing rumen fermentations for enhanced animal production.

In recent years, the search for novel biocatalysts with lignocellulose degradation functionality has gained an utmost attention. Fueled by the recent advancement of ‘omics’ techniques, numerous microbial enzymes have been developed and exploited for various industrial applications. For bio-fuel production as well as other bioconversion processes in paper, textile, food industries, where different treatments such as hot water, steam explosion, alkaline, solvent or acidic pretreatments are employed before or during enzyme treatment, robust enzymes that possess multiple extremophilic traits like thermos-alkaliphilic, thermosacidophilic, or multi-functionality characteristics have the potential to be particularly beneficial players. Earlier investigations by Zhang et al. unveiled a thermostable xylanase sourced from the salt tolerant *Thermobifida halotolerans* strain YIM90462. This enzyme exhibited remarkable xylanase activity at pH 9 and 70 °C, making it a compelling candidate for applications in pulp and paper bioleaching [69]. Additionally, a single fosmid harboring a cellulase enzyme, sourced from the buffalo rumen metagenomic library, exhibited exceptionally high cellulase activity, with its optimal operating conditions at pH 5.5 and 50 °C. This cellulase displayed robust stability under acidic pH conditions, indicating its promising suitability as a potential feed supplement for broiler chicken [70]. In a separate study, Motahar et al. uncovered an acidic-thermostable α-amylase enzyme, PersiAmy2, cloned from the sheep rumen metagenome. The recombinant PersiAmy2 expressed in *E. coli* BL21 (DE3) exhibited remarkable stability under diverse pH, temperature, and maintained its efficacy even in the presence of various ions, inhibitors, and surfactants, which can be promising candidate to enhance the quality of gluten-free bread [71]. Combining metagenome screening with PCR-based methods has resulted in the direct cloning of numerous new genes/enzymes from environmental samples. In this study, we used a sequence-based metagenomics dataset to screen cellulolytic and xylanolytic enzymes from uncultured bacteria in goat rumen fluid. We then cloned and expressed two genes encoding for endoglucanase A and endo-1,4-beta xylanase. The biochemical function of the two enzymes was analyzed by using carboxymethyl cellulose and oat xylan, respectively, as a sole carbon source. This process for characterization of various cellulases and xylanases enzymes from bacterial metagenomes in the goat rumen environment serves as a theoretical framework for better understanding of the regulation of cellulolytic enzyme production.

Multiple alignments of the endoglucnase A from goat rumen bacteria with its homologous proteins with a glycoside hydrolase family 5 (GH5) family domain indicated that they shared only around 51–56% amino acid sequence identity. Likewise, the alignment of the endo-1,4-beta xylanase to its homologous proteins containing a glycoside hydrolase family 10 domain (GH10) showed that they shared only around 57–59% identity similarity [72–74]. Salt bridges between catalytic residues play a vital role in facilitating intramolecular electron transfer (IET) by promoting interactions among catalytic residues and substrate [75]. Notably, the recombinant endoglucanase A gene examined in this study contains nineteen salt bridges. The endo-1,4-beta xylanase was detected with thirteen salt bridges. The occurrence of these salt bridges in various essential regions of the enzyme contributes to its resilience under diverse extreme physicochemical conditions [76]. This revelation underscores the novelty of the enzymes cloned in this investigation, emphasizing that they belong to previously uncharacterized species, indicative of their status as entirely new enzymes characterized by enhanced activity and thermostability. As for optimum pH, and temperature, our findings are consistent with previous studies indicating that the ideal temperature and pH for recombinant endoglucanases produced by cellulolytic rumen bacteria fall within the pH range of 5.0–7.0 and temperature range of 40–50 °C [70, 77–79]. In a previous study, recombinant expression of endoglucanase from *Bacillus licheniformis* ATCC 14580 in *E. coli* BL21 (DE3) resulted in an activity level of 1.5 U/ml under optimized conditions, using carboxymethylcellulose as the substrate [80]. Similarly, another endoglucanase, EG5B, derived from *Paenibacillus sp*. IHB B 3084, was cloned and expressed in *E. coli* BL21(DE3), exhibiting the highest enzymatic activity at 1.382 IU/ml [81]. In both studies, crude enzyme extracts were utilized for enzymatic activity analysis. In contrast, in a separate research endeavor, endoglucanase CenC from *Clostridium thermocellum* was purified before enzyme activity analysis, revealing an activity of 30 U/mg on CMC and 9 U/mg on avicel, respectively [82]. Remarkably, the endo-1,4-beta xylanase obtained from this study exhibited an optimum activity at temperature around 50 °C and pH 10 (within test range). Various previous studies have characterized xylanase enzymes from different sources, including goat rumen [83], marine bacteria [84], camel metagenomes [85], termite gut metagenomes [86], and yak rumen [87]. These xylanases exhibit moderate thermostability and display optimal activity at temperatures around 50–60 °C. Additionally, they tend to have an optimal pH around 8.0 and are functional in a pH range between 5.5 and 8.0. The recombinant xylanase investigated in our study displayed remarkable activity over a wide pH range, making it a promising candidate for industrial processes that demand alkaline conditions. In previous research, the endo-xylanase xynFCB, derived from the thermophilic bacterium *Thermoanaerobacterium saccharolyticum* NTOU1, was subjected to exogenous expression and purification in *E. coli* BL21. This enzyme exhibited its highest activity at 91 U/mg, when oat spelt was employed as the substrate [84]. Similarly, in a separate study, exogenous expression of the endo-β-1,4-xylanase XylH, originating from the gastrointestinal bacterium *Microbacterium trichothecenolyticum* HY-17, revealed optimal xylanolytic activity at a high level of 97 U/mg when oat spelt served as the substrate [88]. While the enzymatic activity analysis conducted in this study did not yield an exceptionally high hydrolysis rate, it's crucial to note that the enzyme preparation process did not incorporate a purification step. Consequently, the crude protein extraction included a mixture of various enzymes, potentially influencing the accuracy of the enzymatic activity evaluation.

## Conclusions

In this study, we have demonstrated the process for investigating and utilizing metagenome resources. The findings from this study highlight the disproportionately significant role that rumen microbes in cellulosic biomass degradation. The in-depth analysis of the goat rumen bacterial metagenomes along with cloning, enrichment enzymatic assay, and in vitro enzyme characterization could serve as a rich resource for the biotechnology community engaged in unearthing novel strategy for lignocellulosic biomass conversion into CH_4_ rich products or other targets. We have demonstrated the process to clone novel genes from the metagenome and producing and characterization of recombinant cellulolytic enzymes. Designing consortia with both anaerobic bacteria and fungi could better aid in understanding the diverse physio-chemical parameters while offering knowledge base to create minimal systems for the bio-chemical conversion of lignocellulose into value added chemicals. While the current study did not assess the relative transcription levels of the identified CAZyme genes, it is worth noting that the microbial consortia detected could potentially encode a substantial number of CAZyme-associated genes that are part of enzyme-tethered systems. Even so, the dataset of goat rumen-derived genomes described in this study, along with publicly available rumen genomes, could serve as a valuable reference for future metagenomic investigations.

### Supplementary Information

**Additional file 1:** **Table S1.** Primers for gene cloning from goat rumen bacterial DNA. **Table S2.** Microbial community analysis using Metaphlan. **Table S3.** Gene counts of CAZymes annotated by DOE-JGI pipelines. **Table S4.** Cellulase and hemicellulase genes deposited into NCBI database with accession number. **Figure S1.** SDS-PAGE analysis of the recombinant proteins. (+) are crude extract of IPTG induced endo 1, 4 beta xylanase (left) around 37kD and endoglucanase A (right) around 38kDa; (+/-) are crude extract with no IPTG induction; (-) are an IPTG induced crude extract of an empty vector (negative control).

## Data Availability

The raw reads were deposited in the NCBI Sequence Read Archive (SRA) under accession number SRX2267715 and SRX2267714. The assembled scaffolds were deposited to the NCBI with accession number VKOM0000000000.1, VKOL000000000.1, and VKOK000000000.1 DOJ-JGI IMG annotation data can be retrieved from https://img.jgi.doe.gov/cgi-bin/m/main.cgi?section=TaxonDetail&page=taxonDetail&taxon_oid=3300001425.

## Abbreviations

CAZymes: Carbohydrate-Active EnZymes
CMC: Carboxymethyl cellulose sodium salt
COG: Cluster of orthologous groups
DNS: Dinitrosalicyclic acid
GH: Glycoside hydrolase
GMQE: Global model quality estimation
IPTG: Isopropyl β-D-1-thiogalactopyranoside
PDB: Protein data bank
SDS-PAGE: Sodium dodecyl sulfate polyacrylamide electrophoresis
SRA: Sequence Read Archive
VMD: Visual molecular dynamics
