# Supplementary material for: Exploring the microbial diversity and characterization of cellulase and hemicellulase genes in goat rumen: a metagenomic approach
Source: BMC Biotechnol. 2023 Dec 4;23:51. doi: 10.1186/s12896-023-00821-6 (PMC10696843; doi:10.1186/s12896-023-00821-6)
Supplement: Supplementary file 1 — Additional file 1: Table S1. Primers for gene cloning from goat rumen bacterial DNA. Table S2. Microbial community analysis using Metaphlan. Table S3. Gene counts of CAZymes annotated by DOE-JGI pipelines. Table S4. Cellulase and hemicellulase genes deposited into NCBI database with accession number. Figure S1. SDS-PAGE analysis of the recombinant proteins. (+) are crude extract of IPTG induced endo 1, 4 beta xylanase (left) around 37kD and endoglucanase A (right) around 38kDa; (+/-) are crude extract with no IPTG induction; (-) are an IPTG induced crude extract of an empty vector (negative control). [file 12896_2023_821_MOESM1_ESM.docx]

**SUPPLEMENTS**

**Exploring the Microbial Diversity and Characterization of Cellulase and Hemicellulase Genes in Goat Rumen: A Metagenomic Approach**

Santosh Thapa^1, 2^, Suping Zhou^1^, Joshua OHair^3^, Kamal Al Nasr^4^, Alexander Ropelewski^5^, Hui Li^1*^

Department of Agricultural and Environmental Sciences, College of Agriculture, Tennessee State University, 3500 John A. Merritt Blvd, Nashville, TN, USA 37209 ^1^

Vanderbilt University Medical Center, 2215 Garland Ave, Nashville, TN, USA 37232 ^2^

Department of Biological Sciences, College of Life & Physical Sciences, Tennessee State University, 3500 John A. Merritt Blvd, Nashville, TN, USA 37209 ^3^

Department of Computer Sciences, College of Engineering, Tennessee State University, 3500 John A. Merritt Blvd, Nashville, TN, USA 37209 ^4^

Pittsburgh Supercomputing Center, 300 S. Craig Street, Pittsburgh, PA, USA 15213 ^5^

Corresponding author^*^ E-mail: [hli@tnstate.edu](mailto:hli@tnstate.edu)

**Table of contents**

Table S1. Primers for gene cloning from goat rumen bacterial DNA.

Table S2. Microbial community analysis using Metaphlan

Table S3. Gene counts of CAZymes annotated by DOE-JGI pipelines.

Table S4. Cellulase and hemicellulase genes deposited into NCBI database with accession number.

Figure S1. SDS-PAGE analysis of the recombinant proteins. (+) are crude extract of IPTG induced endo 1, 4 beta xylanase (left) around 37kD and endoglucanase A (right) around 38kDa; (+/-) are crude extract with no IPTG induction; (-) are an IPTG induced crude extract of an empty vector (negative control).

Table S1. Primers for gene cloning from goat rumen bacterial DNA.

| Gene Name | Forward Primer Sequence | | Reverse Primer Sequence |
| --- | --- | --- | --- |
| Endoglucanase A | | CACCATGCAAGCACAAAGTTTTGAAAC | GTAGAAAGTGATGATGCCATTATTCTT |
| Endoglucanase D | | CACCATGAACGAACCAAGTCTTGCA | ATTGGCGCAAACACCGTA |
| Endoglucanase E | | CACCATGAGGATCAACTGGATGAGAA | TCGCACGACGATCTTTCTTCCA |
| Endo-1,6-beta-D glucanase | | CACCATGAAAATGGGGGAGAACGG | GTAGATTTCCTTGTCCGGTGC |
|  |  |  |  |
| β-glucosidase A | | CACCATGACCTTCCCGGTCAACTTC | TTTACGCGTTACATTGATTTCTTCC |
| Β-xylosidase | | CACCATGCGGGAGTATCTGCCC | CATCCGGGCCGTCAGGATA |
| β_galactosidase | | CACCATGTTGACGAATGGCAAAGG | CAGGTAGATAGGTCTCCGGTTCA |
| α-arabinopyranosidase | | CACCATGCTCCCTAAAAGCTACAGC | GAGGAAATCATCGTCACCGC |
| Xylulose kinase | | CACCATGTGGTGGGACAATGCC | CAGTTTTGCTGCCGTAAAGTTAC |
| Endo-1,4-beta xylanase | | CACCATGAAGGACGTCCAGATCTACATTG | CTCGGCTACCGGAAGTATCTCA |

Table S2 Microbial community analysis using Metaphlan

|  | **phylum** | **class** | **genus** | **species** | **Bct_5121 (%)** | **Bct_5122 (%)** | **Bct_789 (%)** |
| --- | --- | --- | --- | --- | --- | --- | --- |
| Archaea |  |  |  |  | 2.83093 | 3.41544 | 3.12428 |
| Bacteria |  |  |  |  | 97.16907 | 96.58456 | 96.87572 |
| Archaea | Euryarchaeota | Methanobacteria | Methanobrevibacter | Methanobrevibacter_ruminantium | 0.24822 | 0.25514 | 0.19196 |
| Archaea | Euryarchaeota | Methanobacteria | Methanobrevibacter | Methanobrevibacter_smithii | 0.08918 | 0.14159 | 0.18982 |
| Archaea | Euryarchaeota | Methanobacteria | Methanobrevibacter | Methanobrevibacter_unclassified | 2.49353 | 3.01871 | 2.7425 |
| Bacteria | Actinobacteria |  |  |  | 1.58176 | 0.69591 | 1.06028 |
| Bacteria | Actinobacteria | Actinobacteria | Mycobacterium | Mycobacterium_unclassified | 0 | 0 | 0.17389 |
| Bacteria | Actinobacteria | Actinobacteria | Propionibacterium | Propionibacterium_acnes | 0.00854 | 0.07304 | 0.02884 |
| Bacteria | Actinobacteria | Actinobacteria | Propionibacterium | Propionibacterium_unclassified | 0.09124 | 0 | 0.02411 |
| Bacteria | Actinobacteria | Actinobacteria | Bifidobacterium | Bifidobacterium_angulatum | 0 | 0.01566 | 0 |
| Bacteria | Actinobacteria | Actinobacteria | Bifidobacterium | Bifidobacterium_longum | 0.42775 | 0 | 0 |
| Bacteria | Actinobacteria | Actinobacteria | Bifidobacterium | Bifidobacterium_unclassified | 0.26035 | 0.10639 | 0.20431 |
| Bacteria | Actinobacteria | Actinobacteria | Olsenella | Olsenella_uli | 0 | 0 | 0.01239 |
| Bacteria | Actinobacteria | Actinobacteria | Slackia | Slackia_heliotrinireducens | 0.45418 | 0.45792 | 0.40788 |
| Bacteria | Actinobacteria | Actinobacteria | Slackia | Slackia_unclassified | 0.3397 | 0.0429 | 0.20886 |
| Bacteria | Bacteroidetes |  |  |  | 24.56877 | 26.66641 | 28.52052 |
| Bacteria | Bacteroidetes | Bacteroidia | Bacteroides | Bacteroides_unclassified | 2.43564 | 2.89147 | 5.22288 |
| Bacteria | Bacteroidetes | Bacteroidia | Prevotella | Prevotella_bergensis | 0 | 0 | 0.04652 |
| Bacteria | Bacteroidetes | Bacteroidia | Prevotella | Prevotella_bryantii | 0 | 0.01115 | 0 |
| Bacteria | Bacteroidetes | Bacteroidia | Prevotella | Prevotella_buccae | 0 | 0.04152 | 0.13136 |
| Bacteria | Bacteroidetes | Bacteroidia | Prevotella | Prevotella_copri | 0 | 0.00455 | 0.00807 |
| Bacteria | Bacteroidetes | Bacteroidia | Prevotella | Prevotella_marshii | 0 | 0 | 0.00653 |
| Bacteria | Bacteroidetes | Bacteroidia | Prevotella | Prevotella_multiformis | 0 | 0.01052 | 0.03665 |
| Bacteria | Bacteroidetes | Bacteroidia | Prevotella | Prevotella_ruminicola | 22.11236 | 23.43675 | 22.63532 |
| Bacteria | Bacteroidetes | Bacteroidia | Alistipes | Alistipes_unclassified | 0.02078 | 0.07687 | 0.3163 |
| Bacteria | Bacteroidetes | Sphingobacteria | Sphingobacteriaceae_unclassified |  | 0 | 0.19357 | 0.11687 |
| Bacteria | Chlamydiae | Chlamydiae | Chlamydiaceae_unclassified |  | 0 | 0 | 0.06529 |
| Bacteria | Chloroflexi | Thermomicrobia |  |  | 0 | 0.03068 | 0.05446 |
| Bacteria | Fibrobacteres | Fibrobacteres | Fibrobacter | Fibrobacter_succinogenes | 16.47777 | 15.86824 | 14.10563 |
| Bacteria | Firmicutes |  |  |  | 48.67042 | 47.76667 | 45.01231 |
| Bacteria | Firmicutes | Clostridia | Pseudoflavonifractor | Pseudoflavonifractor_capillosus | 0 | 0.00523 | 0.03079 |
| Bacteria | Firmicutes | Clostridia | Clostridiales_Family_XI_Incertae_Sedis_unclassified |  | 0 | 0.0168 | 0 |
| Bacteria | Firmicutes | Clostridia | Blautia | Blautia_unclassified | 0.07729 | 0.2077 | 0.22848 |
| Bacteria | Firmicutes | Clostridia | Butyrivibrio | Butyrivibrio_fibrisolvens | 0.28107 | 0.35174 | 0.37054 |
| Bacteria | Firmicutes | Clostridia | Butyrivibrio | Butyrivibrio_proteoclasticus | 5.82036 | 6.21607 | 5.39922 |
| Bacteria | Firmicutes | Clostridia | Butyrivibrio | Butyrivibrio_unclassified | 39.65145 | 37.92736 | 35.86064 |
| Bacteria | Firmicutes | Clostridia | Faecalibacterium | Faecalibacterium_unclassified | 0 | 0 | 0.01472 |
| Bacteria | Firmicutes | Clostridia | Ruminococcus | Ruminococcus_albus | 1.86932 | 2.12341 | 1.98744 |
| Bacteria | Firmicutes | Clostridia | Ruminococcus | Ruminococcus_flavefaciens | 0.59363 | 0.60466 | 0.54919 |
| Bacteria | Firmicutes | Negativicutes | Acidaminococcaceae_unclassified |  | 0.14946 | 0.1789 | 0.24303 |
| Bacteria | Firmicutes | Negativicutes | Mitsuokella | Mitsuokella_multacida | 0.00702 | 0.0359 | 0.03615 |
| Bacteria | Firmicutes | Negativicutes | Selenomonas | Selenomonas_unclassified | 0.09707 | 0.0989 | 0.29211 |
| Bacteria | Firmicutes | Negativicutes | Veillonella | Veillonella_parvula | 0.12377 | 0 | 0 |
| Bacteria | Proteobacteria |  |  |  | 5.717 | 5.54066 | 8.03803 |
| Bacteria | Proteobacteria | Alphaproteobacteria | Methylobacterium | Methylobacterium_radiotolerans | 0 | 0.0016 | 0 |
| Bacteria | Proteobacteria | Alphaproteobacteria | Methylobacterium | Methylobacterium_unclassified | 0 | 0.07358 | 0 |
| Bacteria | Proteobacteria | Alphaproteobacteria | Acetobacteraceae_unclassified |  | 0 | 0 | 0.16427 |
| Bacteria | Proteobacteria | Deltaproteobacteria | Desulfobulbaceae_unclassified |  | 0.52867 | 1.15003 | 1.96312 |
| Bacteria | Proteobacteria | Deltaproteobacteria | Desulfovibrio | Desulfovibrio_desulfuricans | 5.14961 | 4.26735 | 5.60502 |
| Bacteria | Proteobacteria | Gammaproteobacteria | Escherichia | Escherichia_coli | 0 | 0 | 0.28051 |
| Bacteria | Proteobacteria | Gammaproteobacteria | Mannheimia | Mannheimia_haemolytica | 0.03871 | 0.0481 | 0.02512 |
| Bacteria | Synergistetes | Synergistia | Pyramidobacter | Pyramidobacter_piscolens | 0 | 0.016 | 0.0192 |
| Bacteria | Verrucomicrobia | Verrucomicrobiae | Akkermansia | Akkermansia_muciniphila | 0.15335 | 0 | 0 |

Table S3. Gene counts of CAZymes annotated by DOE-JGI pipelines.

| COG ID | COG Name | Gene Count |
| --- | --- | --- |
| Cellulose Degradation | | |
| COG3405 | Endo-1,4-beta-D-glucanase-Y | 347 |
| COG5309 | Exo-beta-1,3-glucanase,GH17 | 14 |
| COG5498 | Endoglucanase Acf2 | 58 |
| COG2273 | Beta-glucanase, GH16 | 607 |
| COG5297 | Cellulase/cellobiase CelA1 | 26 |
| COG2723 | Beta-glucosidase/6-phosphobeta-glucosidase | 1579 |
| Hemicellulose Degradation | | |
| COG3867 | Arabinogalactan endo-1,4-betagalactosidase | 1634 |
| COG3534 | Alpha-L-arabinofuranosidase | 3115 |
| COG3693 | Endo-1,4-beta-xylanase, GH35 | 1753 |
| COG3664/3507 | Beta xylosidase | 4475 |
| COG3940 | Beta-xylosidase, GH43 | 266 |
| COG3250 | Beta-galactosidase/betaglucuronidase | 10920 |

Table S4. Cellulase and hemicellulase genes deposited into NCBI database with accession number.

|  | Gene Name | NCBI Accession Number^#^ |
| --- | --- | --- |
| 1. | Endo 1, 4 beta xylanase | KP851788 |
| 2. | Endoglucanase A | KP851789 |
| 3. | Beta-glucosidase A | KP851790 |
| 4. | Endo-1,6-beta-D-glucanase | KP851791 |
| 5. | Endoglucanase E | KP851792 |

Note: The five genes were cloned into E. coli Top10. Gene sequences were confirmed by Sanger sequencing of plasmid extraction.


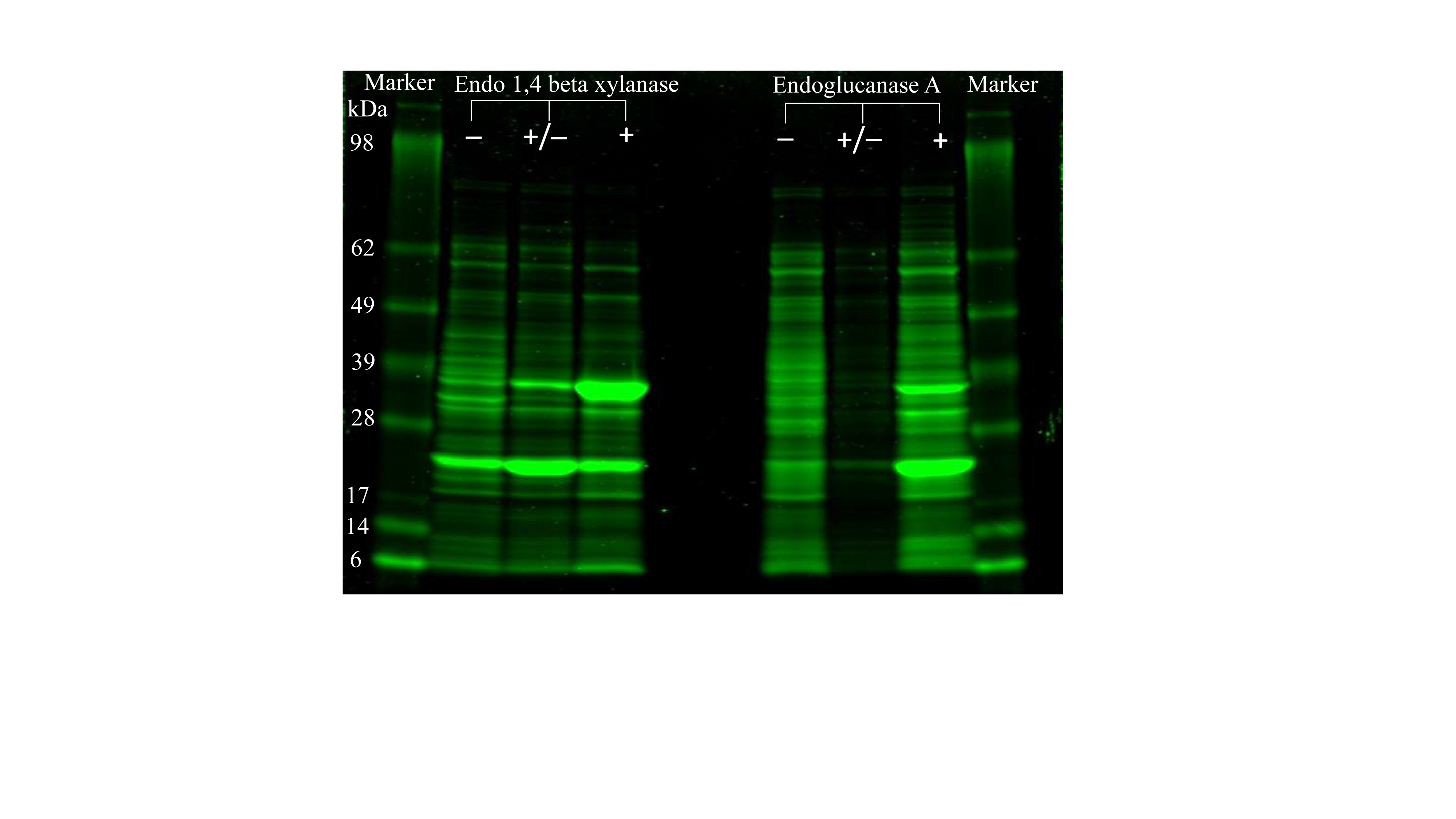


Figure S1. SDS-PAGE analysis of the recombinant proteins. (+) are crude extract of IPTG induced endo 1, 4 beta xylanase (left) around 37kD and endoglucanase A (right) around 38kDa; (+/-) are crude extract with no IPTG induction; (-) are an IPTG induced crude extract of an empty vector (negative control).
